# Drug therapy problems and treatment satisfaction among ambulatory patients with epilepsy in a specialized hospital in Ethiopia

**DOI:** 10.1371/journal.pone.0227359

**Published:** 2020-01-03

**Authors:** Beshir Bedru Nasir, Alemseged Beyene Berha, Meron Awraris Gebrewold, Yared Mamushet Yifru, Ephrem Engidawork, Minyahil Alebachew Woldu

**Affiliations:** 1 Department of Pharmacology and Clinical Pharmacy, School of Pharmacy, College of Health Sciences, Addis Ababa University (AAU), Addis Ababa, Ethiopia; 2 Department of Neurology, School of Medicine, College of Health Sciences, AAU, Addis Ababa, Ethiopia; University of Catanzaro, ITALY

## Abstract

**Objective:**

Epilepsy management especially in developing country is challenging. Seizures recurrence can be caused by both drug and non-drug related problems such as inadequate antiepileptic regimens, adverse drug reaction and poor adherence. Patient treatment satisfaction also affects the treatment out comes by improving medication adherence. This study aimed to assess drug therapy problems (DTPs) and treatment satisfaction among ambulatory epileptic patients at Tikur Anbessa Specialized Hospital.

**Methods:**

A prospective cross-sectional study was conducted on 291 epileptic patients. Data was collected through patient interview and medical charts review. DTPs were identified based on the standard treatment guidelines and Micromedex® was used as drug interaction checker. Cipolle DTPs classification was used to classify the DTPs and Treatment Satisfaction with Medicine Questionnaire (SATMED-Q) was used to assess treatment satisfaction. Binary logistic regressions were utilized to identify the associated factors.

**Results:**

Phenobarbital 195 (67%) and phenytoin 97 (33.3%) were the most frequently prescribed antiepileptic medications as monotherapy or combination therapy. Only 54 (18.6%) of the study participants had controlled seizure. DTP was found in 205(70.4%) of the study participants. From 352 DTPs identified, adverse drug reaction 146 (41.5%) was the leading DTPs followed by ineffective drugs 98 (27.8%) drug interaction 45 (12.8%) and inappropriate dose 42(11.9%). Headache, depression and epigastric pain were frequently reported adverse drug reaction. Among the study participants 167 (57.3%) were adherent to their medications. The number of medications taken by the patients had significant association with occurrence of DTPs, whereas source of medication and seizure free periods were found to have significant association with poor adherence. The global patient satisfaction was (67.4%) and lower satisfaction rate was found with regard to impact on daily activities (62.0%), treatment effectiveness (64.7%) and medical care (65.9%).

**Conclusion:**

Prevalence of DTPs among ambulatory epileptic patients was high and about half of the patients were nona-dherent for their medication. The overall treatment satisfaction of the patients was suboptimal.

## Introduction

Epilepsy is a neurologic disorder characterized by an enduring predisposition to generate epileptic seizures [1]. In epilepsy treatment, the ultimate goal is to prevent seizure episode without side effects and having an optimal quality of life [2]. The management should therefore be individualized to eliminate or reduce seizure frequency, while avoiding drug-related harms and complications [3, 4]. However, about 90% of epileptic patients in developing countries are not receiving appropriate treatment due to cultural attitudes, lack of prioritization, poor health care system and inadequate supply of antiepileptic drugs (AEDs) [5]. Moreover, pharmacokinetics of most AEDs is complex, which makes dosing and monitoring very difficult. Complexity of medical problems and co-medications given with AEDs can increase drug-related problems (DRPs) & drug interactions, which can affect seizure control and toxicity [6]. Enzyme-inducing AEDs such as carbamazepine (CBZ) may accelerate the metabolism of many drugs including antiretroviral, anti-tuberculosis (anti-TB), and hormonal contraceptives thereby reducing their concentration by up to 50% [7].

DTP is any undesirable event experienced by a patient that involves or suspected to involve drug therapy, and that interferes with achieving the desired goals of therapy. An infinite number of DTPs exist because of rapidly expanding array of drugs available, increasing number of diseases being diagnosed and number of patients entering the healthcare system [8].

AEDs-related DTP studies are sparse in the literature. The studies so far made are either on utilization or adherence of AEDs. AEDs utilization study carried out in Bishoftu hospital, Ethiopia, identified under dosing (16.5%), incorrect duration (12.7%), and drug-drug interactions (5%) to be the major therapy related problems [9]. Non-adherence to medication accounts for substantial worsening of disease, death and increased health care costs [10]. Previous studies regarding AEDs adherence produced varied results. Adherence was higher in India (98.6%) [11] and Palestine (85.3%) [12]. It was relatively lower in China (51.9%) [13], Jimma hospital, Ethiopia (58.5%) [10] and the UK (59%) [14]. Moderate adherence was reported from the USA (71%) [15] and Gondar hospital, Ethiopia (70.8%) [16]. Several lines of evidence indicate that adherence to AEDs is suboptimal [17]. Collectively, these reports suggest that identification of common DTPs is an important component of drug therapy and contributes to reduction of drug-related morbidity and mortality.

Regarding consequences of low treatment satisfaction, one half of patients with chronic illness end up making medication related decisions without seeking medical advice, becoming non-adherent that may compromise the effectiveness of treatment. Furthermore, patients having better satisfaction on the treatment are more tend to adhere their medication and improve the treatment out comes [18].

Therefore, this study was carried out to determine the prevalence of DTPs and associated factors and treatment satisfaction among epileptic ambulatory patients in a tertiary care teaching hospital of Ethiopia.

## Material and Methods

### Study design and population

A prospective cross-sectional study was conducted among ambulatory epileptic patients at neurology clinic of Tikur Anbessa Specialized Hospital (TASH), which is the largest referral and teaching hospital in the country. Patients having epilepsy, had complete medical records and willing to participate in the study were included. Data were collected from 4^th^ June to 28^th^ August 12, 2017.

### Sampling

Sample size was computed based on single population proportion formula. The total epileptic patients at TASH were 1100. Using correction formula, 291 patients were recruited by systematic random sampling. Pretest was conducted on 5% of the sample size to ensure relevance of the instruments and appropriate modifications were performed accordingly.

### Data collection

Patient information was collected through interview and chart review by trained nurses and clinical pharmacists, respectively. The relevant information about each patient (demographic data, patients’ clinical characteristics and patients’ treatment satisfaction) was collected from patient interview following neurology clinic visit. Laboratory results, current medications and co-morbidities were collected from patients’ medical chart.

Appropriateness of medical therapy was evaluated using guidelines of National Institute for Health and Care Excellence, American Academy of Neurology, and Ethiopian treatment guideline for general hospitals 2014. Micromedex® (Micromedex 2.0. Truven Health Analytics Inc.) drug interaction checker was used to identify drug interactions and only absolute contraindications and major drug interactions were considered as significant interactions. The identified DTPs were classified using DTP registration format adopted from Cipolle *et al*. [16] with slight modification. Adherence was measured using Morisky Medication Adherence (Morisky Green Levine test[19] which is online available for free http://www.pmidcalc.org/?sid=3945130&newtest=Y). Patients’ treatment satisfaction was assessed by using treatment Satisfaction with Medicines Questionnaire (SATMED-Q) which is composed of 17 items investigating 6 dimensions (undesirable side effects, treatment effectiveness, convenience to use, impact on daily activity, medical care and global satisfaction.

### Data analysis

Data were entered using Epi Info version 7.2.1 and analysed with Statistical Package for Social Science version 21. Descriptive statistics was used to summarize patients’ characteristics. Tables and charts were used to present the findings. Prevalence of DTP was calculated by dividing the number of patients who had at least one DTP with the study participants. Multivariable binary logistic regression analysis was used to assess association of the independent variables with DTPs and medication adherence after univariable analysis (p<0.2) to control confounders. P value < 0.05 was considered statistically significant. Regarding treatment satisfaction, the sum of the direct score of each dimension was changed to a more intuitive and easier to understand metric with a minimum of 0 and a maximum of 100%.

### Ethics approval

The study was approved by Institutional Review Board of College of Health Sciences, Addis Ababa University (Protocol number = 002/17/SPharma). A written informed consent was obtained from all study participants before the data collection.

## Results

### Socio-demographic characteristics

The mean age was 30.2 ±11.4 years and about half of them 154(50.2%) were young adults. Majority of them were single 185(63.6%) and from Addis Ababa 238(81.1%). Around 105(36%) had secondary school education, while 16(5.5%) had no formal education. Almost half 140(48.1%) of the patients got their medication for free (Table 1).

**Table 1:** Socio demographic characteristics of epileptic patients attending at the neurology clinic of TASH, 2017.

**Variables**		**Number (n)**	**Percentage (%)**
**Sex**	Male	154	52.9
	Female	137	47.1
**Age**	Adolescent (<18)	39	13.4
	Young adult (18–30)	146	50.2
	Adult (31–60)	99	34.0
	Elderly (>60)	7	2.4
**Marital status**	Single	185	63.6
	Married	97	33.3
	Divorced	5	1.7
	Widowed	4	1.4
**Residential area**	Addis Ababa	238	81.8
	Out of Addis Ababa	53	18.2
**Educational status**	No formal	16	5.5
	Primary	88	30.2
	Secondary	105	36.1
	Tertiary	82	28.2
**Occupation**	Unemployed	112	38.5
	Employed	69	23.7
	Private/ merchant	49	16.8
	Student	50	17.2
	Daily labourer	2	0.7
	Farmer	6	2.1
	Others*	3	1.0
**Source of medication**	Free	140	48.1
	Payment	151	51.9

* retired

#### Clinical characteristics

Generalized tonic clonic seizure (GTCS) was the commonest 193(66.3%) seizure type among the participants and 191(65.6%) had seizure free period less than a year. About 116(40%) had 1–5 seizure episodes per year and 35(12.7%) of them had more than ten seizure episode in the previous one year. Only 54 (18.6%) patients had controlled seizure (seizure free period of two years or longer). Almost a quarter (23.4%) of them had chronic comorbid conditions, HIV infection and hypertension being the commonest ones (Table 2).

**Table 2:** Clinical characteristics of epileptic patients attending at the neurology clinic of TASH, 2017.

Variables			Number (n)	Percentage (%)
**Types of seizure**	***Generalized seizure***		197	67.7
		GTCS	193	66.3
		Myoclonic	3	1.0
		Absence	1	0.3
	***Partial seizure***		71	24.4
		Simple partial	19	6.5
		Complex partial	23	7.9
		Focal with secondary generalization	29	10.0
	Unclassified*		23	7.9
**Follow up years**	< 2 years		49	16.8
	2–5 years		53	18.2
	6–10 years		69	23.7
	>10 years		120	41.2
**Seizure free period**	< 1 year		191	65.6
	1–2 years		46	15.8
	2–5 years		33	11.3
	>5 year		21	7.7
**Number of seizure episode per year**	None		102	35.1
	1–5		120	41.2
	6–10		32	11.0
	>10		37	12.7
Co-morbid conditions	HIV		20	6.9
	CRVHD/CHF		8	2.7
	Major depressive disorder		10	3.4
	Migraine headache		3	1.0
	Mental retardation		5	1.7
	HTN		12	4.1
	Stroke		7	2.4
	Others		14	4.8

“*Unclassified seizure”, a seizure which is diagnosed by the physician as “Epilepsy” or “seizure disorder”; GTCS, Generalized Tonic Clonic Seizure; HIV, human immune virus; CHF, Congestive Heart Failure; CRVHD, Chronic Rheumatic Valvular Heart Disease.

### Pattern of antiepileptic drug use

Regarding AEDs use, monotherapy was the common (58.8%) mode of therapy and phenobarbital 93(32%) and phenytoin 32(92%) were the drug of choices. Dual and triple therapy accounted for 33.3% and 7.2%, respectively. Phenobarbital + phenytoin 30(10.3%), phenobarbital + carbamazepine 17(5.6%), and Phenobarbital + valproic acid 15(5.3%) were the most frequently used dual therapy regimen. One fourth (25.1%) of the study participants were taking more than two medications, with mean and SD of 2.03±1.27 medications per day (Table 3).

**Table 3:** Pattern of medication use among epileptic patients attending at the neurology clinic of TASH, 2017.

Variables		Number (N)	Percentage (%)
**Number of AEDs used**	One	171	58.8
	Two	97	33.3
	Three	21	7.2
	Four	2	0.7
**AEDs use Pattern**	PHB	93	32.0
	PHT	35	12.0
	CBZ	32	11.0
	VPA	17	5.8
	LTG	1	0.3
	PHB+ PHT	30	10.3
	PHB+CBZ	17	5.6
	PHB+VPA	15	5.2
	PHB+LTG	1	0.3
	PHT+CBZ	13	4.5
	PHT+VPA	9	3.1
	CBZ+VPA	7	2.4
	PHB+PHT+CBZ	4	1.4
	CBZ+VPA+PHB	4	1.4
	PHB+PHT+VPA	1	0.3
	PHB+PHT+CLONA	1	0.3
	Others *	11	3.8
**Total number of** **drugs (AEDs +** **others)**	1	120	41.2
	2	97	33.3
	>2	74	25.5

Others *, different combination of the above AEDs including quadruple therapy; PHB, phenobarbital; PHT, phenytoin; CBZ, carbamazepine; VPA, Valproic acid; LTG, Lamotrigine; CLONA, Clonazepam.

Analysis of drug use based on seizure type revealed that while phenobarbital and phenytoin were frequently used for generalized seizure, carbamazepine and phenobarbital for partial seizure. Phenobarbital was also frequently used for unclassified seizure and the use of valproic acid for any seizure was low.

### Drug therapy problems

A total of 352 DTPs were identified from 205 participants, giving rise to a prevalence of 70.4%. One DTP was identified in 102 (49.8%), 2 DTPs in 73 (35.6%) and >2 DTPs in 30 (14.6%) participants. Average number of DTPs per patient was 1.2 ± 0.4. Adverse drug reactions (ADRs) followed by inappropriate drug selection and drug interaction were the major DTPs found in the present study (Table 4). DTPs appeared to be common in patients with uncontrolled seizure (r = -0.264, P = 0.000). The proportion was 76% for uncontrolled and 52% for controlled seizure.

**Table 4:** Types of drug therapy problems identified from epileptic patients attending at the neurology clinic of TASH, 2017.

**DTPs**		**No. of DTPs**	**Total**	**(%)**
**Drug interaction**		45	45	12.8
**Adverse drug reaction**	Undesired effect	144	146	41.5
	Contraindications	2		
**Ineffective drugs**	Inappropriate drug selection	80	98	27.8
	Condition refractory to the drug	18		
**Need additional therapy**	Untreated medical condition	13	18	5.1
	Need synergistic/potentiating	5		
**Inappropriate dose**	Dose too low	24	42	11.9
	Dose too high	18		
**Unnecessary drug therapy**	No medical indication	2	3	0.9
	Duplicate therapy	1		
**Total**			352	100

DTP: Drug Therapy Problems

**Fig 1:**
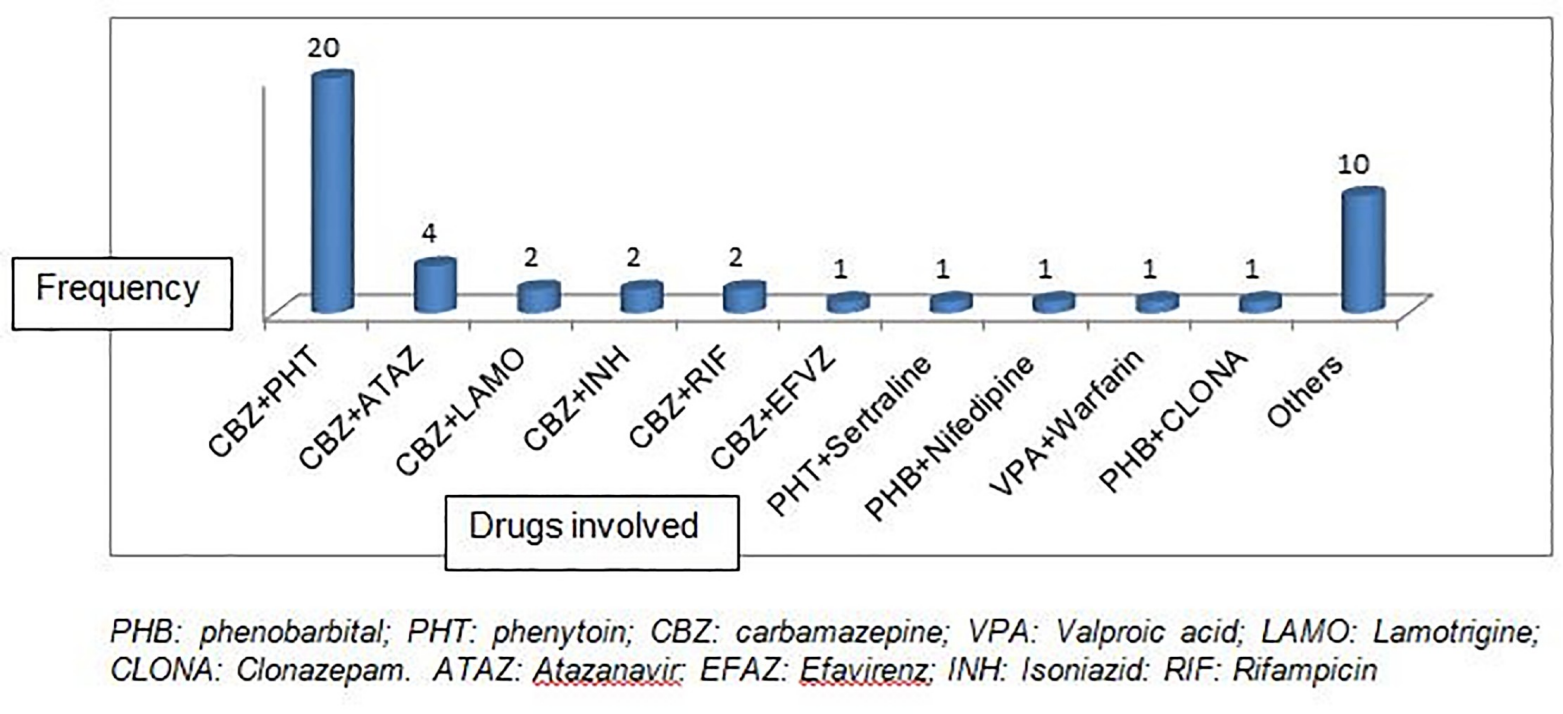
Type of drugs involved in drug interaction and their frequency.

A total of 45 significant drug interactions were identified in 38 (13.1%) study participants and carbamazepine was the culprit in majority of the cases (Fig 1).

One hundred forty four 144(49.5%) of the participants experienced at least one ADR that could possibly be associated with AEDs. Headache 39(13.4%), depression 36(12.4%), epigastric pain 35(12%), and hypersomnia 28(9.6%), were the top four ADRs reported.

### Medication adherence and possible reasons for non-adherence

Adherence was found to be 55.7%. The major reason for poor adherence was forgetfulness followed by patient feeling as cured, medication unavailability, patient belief on drug effectiveness, fear of side effects, and switching to traditional medicines.

### Predictors of occurrence of drug therapy problems

Number of medications was shown to be a risk factor for DTPs. Patients who were on more than 2 medications were 3.8 times more likely to develop DTPs as compared on single medication (AOR = 3.810 CI 1.409–10.30) (Table 5).

**Table 5:** Predictors of occurrence of drug therapy problems in epileptic patients attending at neurology clinic of TASH, 2017.

**Variables**	**category**	**DTPs (%)**		**COR** **(95% CI)**	**AOR** **(95% CI)**	**P-Value**
		Yes	No			
**Gender**	Male	106(73.6)	48(32.7)	1.00	1.00	
	Female	38(23.6)	99(67.3)	1.18(0.711–1.957)	1.19(.667–2.13)	0.552
**Age**	<18	33(16.1)	6(7.0)	1.00	1.00	
	18–30	104(50.7)	42(48.8)	0.450(0.176–1.153)	0.371(0.129–1.068	0.066
	30–60	65(31.7)	34(39.5)	0.348(0.133–0.911)	0.256(0.089–0.790	0.057
	>60	3(1.5)	4(4.7)	0.136(0.024–0.770)	0.135(0.017–1.081	0.059
**Number of medications**	1	73(35.6)	47(54.7)	1.00	1.00	
	2	71(34.6)	26(30.2)	1.758(0.985–3.140)	1.350(0.687–2.652)	0.383
	>2	61(29.8)	13(15.1)	3.273(1.594–6.719)	3.810(1.409–10.30)	0.008*
**Co-morbidities**	No	155(75.6)	68(79.1)	1.00	1.00	
	Yes	50(24.4)	18(20.9)	1.22(0.662–2.242)	1.475(0.286–1.607)	0.378

COR, crude odds ratio; AOR, adjusted odds ratio; CI, confidence interval

### Predictors of non-adherence

Source of medication and seizure free period were shown to be predictors of non-adherence. The odds of being non-adherent was higher by 2.3 fold (AOR = 1.360–3.943) for patients getting medication for free than paying ones. Non-adherence significantly decreased by 64% and 54% in patients with seizure free period of 1–2 years and 2–5 years, respectively, as compared to those with below one year (Table 6).

**Table 6:** Determinant factors associated with medication adherence among epileptic patients attending at neurology clinic of TASH, 2017.

**Variable**	**Category**	**Adherence N (%)**		**COR** **(95% CI)**	**AOR** **(95% CI)**	**P-Value**
		Non adherent	Adherent			
**Educational status**	No formal	4 (3.1)	12 (7.4)	1.00	1.00	
	Primary	40(31.0)	48(29.6)	2.500(0.748–8.358)	2 .848(0. 824–9.839)	0.098
	Secondary	46(35.7)	59(36.4)	2.339(0.708–7.730)	3.266(0. 939–11.355)	0.063
	Tertiary	39(30.2)	43(2 6.6)	2.721(0.810–9.141)	5.177(1.412–18.584)	0.013
**Seizure free years**	< 1	98(76.0)	93(57.4)	1.00	1.00	
	1–2	14(10.9)	32(19.8)	0.415(0.208–0.827)	0.359 (0.170–0.761)	0.007*
	2–5	11(8.5)	22(13.6)	0.474(0.218–1.032)	0.455 (0.198–1.044)	0.043*
	>5	6(4.6)	15(9.2)	0.380(0.141–1.020)	0.368 (0.129–1.050)	0.062
**Source of medication**	Paying	55(42.6)	96(59.3)	1.00	1.00	
	Free	74(57.4)	66(40.7)	1.957(1.224–3.128)	2.316(1.360–3.943)	0.002*
**Number of mediations**	1	45(34.9)	75(46.3)	1.00	1.00	
	2	45(34.9)	52(32.1)	1.442(0.837–2.485)	1.295(0.731–2.298)	0.098
	>2	39(30.2)	35(21.6)	1.912(1.060–3.449)	1.728(0.939–11.355)	0.063
**Occurrence of ADRs**	No	60(46.5)	87(53.7)	1.00	1.00	
	Yes	69(53.5)	75(46.3)	1.334(0.839–2.121)	1.273(0.784–2.080)	0.327

COR, crude odds ratio; AOR, adjusted odds ratio; CI, confidence interval

### Patients’ treatment satisfaction among the study participants

The global satisfaction was 67.4% and it was above 50% with regard to all the six dimensions (Table 7).

**Table 7:** Patients’ treatment satisfaction among epileptic patients attending at neurology clinic of TASH, 2017.

**SATMED-Q dimension**	**Satisfaction score (Mean ± SD)**
Undesirable side effects (0–100)	87.1 ± 20.9
Treatment effectiveness (0–100)	64.7 ± 21.9
Convenience of use (0–100)	72.7 ± 17.0
Impact on daily activities (0–100)	62.0 ± 23.3
Medical care (0–100)	65.9 ± 20.7
Global satisfaction (0–100)	67.4 ± 17.5
Total composite score (0–100)	70.2 ± 12.5

**SATMED-Q**: Satisfaction with Medicines Questionnaire

## Discussion

GTCS was found to be the commonest seizure type encountered, which is in line with studies conducted in Saudi Arabia (65%) [20] and Bangladesh (74%) [21]. Choice of the most appropriate AEDs depends on proper classification of seizures type. Lack of proper seizure classification affects AEDs selection and treatment outcome [22, 23]. About 8% of the study participants had uncategorized seizure type, which could possibly have contributed to inappropriate drug selection and poor treatment outcomes. Monotherapy was the preferred treatment modality and this is in concordance with some [23] but discordant with others [9]. The reason for the discrepancy could be attributed to settings, as this was conducted in a tertiary hospital, where complicated and uncontrolled seizures are referred that might need combination therapy.

Although phenobarbital is not recommended as a first choice for any seizure type, it was the most frequently prescribed agent both as monotherapy and combination therapy. In other studies, agents like phenytoin [11], valproic acid [20], and carbamazepine [21] were the most commonly used ones. The use of phenobarbital despite the recommendation might be associated with cost issues. The study demonstrated that treatment was suboptimal, as the rate of seizure free period of less than a year was quite high (65.6%). This is similar with reports coming out from other developing countries [5] and could be attributed to DTPs like non-adherence and inappropriate selection of AEDs. This notion could be reinforced by the high rate of use of phenobarbital as a preferred agent.

About 70% of the study participants had at least one DTP, in which ADR (41.5%) was the most frequently encountered DTP. Several studies have identified ADR as the commonest problem, with a higher (82%) [24] or lower (31%) [25] rate than the present report that could probably related to methodological differences. Moreover, similar ADR profiles to this report have also been identified in other studies conducted in Ethiopia, [9]indicating action should be taken to relegate phenobarbital.

The second most common DTP identified was in-effective drug (27.8%). According to Cipolle *et al*. [24] DTP classification, this category has two major components (inappropriate drug selection and condition is refractory to the drug). Inappropriateness is commonly due to drug selection problem with regard to seizure type. For example, patients were taking phenobarbital for focal seizure, while phenytoin and carbamazepine are first line agents according to guidelines. Another example is the use of narrow spectrum AEDs for unclassified seizure type instead of valproic acid, as it is the AEDs of choice for unclassified seizure. The reasons for high rate of ineffective drug use might be drug cost, availability problems and lack of local treatment guidelines for tertiary hospital.

Carbamazepine was the most frequently involved AED in drug-interaction due to its potent liver enzyme inducing properties. Generally, AEDs have a wide range of drug interactions among themselves and with other drugs due to enzyme induction/inhibition or competition for plasma protein binding. Such drug interaction might cause ADRs, therapeutic failure and drug-related harm to patients [7]. Carbamazepine’s interactions with anti-TB and antiretrovirals could likely cause treatment failure or drug resistance. Therefore, changing the medication or dose adjustment is recommended. Furthermore, the interaction of phenobarbital with nifedipine, and valproic acid with warfarin could also have resulted in uncontrolled hypertension and increase in INR value, respectively. Potential drug-drug interactions should be ruled out prior to initiating AEDs.

The principle of seizure treatment is starting with low dose and escalating to a maximum dose based on treatment response and tolerability. The rate of inappropriate dosing was 11.9%, with phenobarbital and carbamazepine administered with high and low dose, respectively. Since most of the neurologic side effects are dose-related, AEDs don’t have acceptable safety profile. Patients on high dose of AEDs should therefore be monitored for toxicity and determining serum concentration is required for patients with uncontrolled seizure.

The most common untreated conditions in this study were epigastric pain, migraine headache and anaemia. Epigastric pain is a cause for poor adherence and its management could help to improve quality of life and medication adherence. Identification of risk factors for DTPs is helpful in finding patients at risk and taking action to avoid overt DTPs. This study demonstrated that the number of medications taken by a patient was an important risk factor for DTPs (Table 5) and this is supported also by other studies [26, 27].

AEDs adherence was 55.7%, which is similar with other studies in Ethiopia (58.5%) [10], China (51.9%) [13] and Saudi Arabia (62.7%) [20]. The rate was, however, lower than Palestine (85.3%) [12], India (98.6%) [11] and the USA (71%) [15]. The discrepancies could be due to differences in culture, belief, education and physicians approach to their patients. Source of medication was significantly associated with poor adherence. Free patients were 2.3 times more non-adherent than paying patients (Table 6). This might be due to the fact that medication supply in the hospital was inadequate and most free patients couldn’t afford to buy from outside. The findings also showed that seizure free year was associated with adherence of patients. Longer seizure free years would make patients to be more adherent to their medications possibly by increasing patients’ belief on medication effectiveness and this is in line with other studies [13].

The present study revealed that, global satisfaction of (67.4%) similar with the finding of Palestine (68.4%) [12]. Patient satisfaction with regard to impact of the medication therapy on daily activity was only 62.0%. This might be due about 38.5% of the participants are unemployed and treatment with AEDs restrict them from some daily activities like working with machineries. The satisfaction on treatment effectiveness (64.7%) and medical care (65.9%) was also low. The reasons could be most (65.6%) of the study participants had a seizure free period below one year and high number of patient flow that will compromise the quality of medical care.

The cross-sectional nature of the study and self-reported measure of adherence that could lead to overestimation of adherence could be among limitations of the study. Nevertheless, the optimal sample size, randomized sampling techniques from a tertiary hospital, use of both patient interview and medical chart review could offset the limitations and enhance generalizability the study.

## Conclusion

The findings clearly indicate that there is a high prevalence of DTPs, which could negatively affect management of epileptic patients. The problems largely emanated from inefficient supply system, absence of clinical pharmacy services, poor patient health education, and lack of tailored guideline. Consequently, the overall patients’ treatment satisfaction was suboptimal. Measures should therefore be taken to circumvent these problems so that patients could enjoy seizure free lives.

## Supporting information

S1 file Data collection tool. (DOCX)

## Abbreviations

ADR: Adverse Drug Reaction
AEDs: Anti-Epileptic Drugs
AOR: Adjusted Odds Ratio
CBZ: Carbamazepine
CLONA: Clonazepam
COR: Crude Odds Ratio
DRPs: Drug Related Problems
DTPs: Drug Therapy Problems
GTCS: Generalized Tonic Clonic Seizure
LTG: Lamotrigine
MMAS: Morisky Medication Adherence Scale
PHB: Phenobarbital
PHT: Phenytoin
SATMED-Q: Scaling and Scoring of the Treatment Satisfaction with Medicines Questionnaire
TASH: Tikur Anbessa Specialized Hospital
VPA: Valproic acid
