# Supplementary material for: Drug therapy problems and treatment satisfaction among ambulatory patients with epilepsy in a specialized hospital in Ethiopia
Source: PLoS One. 2020 Jan 3;15(1):e0227359. doi: 10.1371/journal.pone.0227359 (PMC6941812; doi:10.1371/journal.pone.0227359)
Supplement: S1 file — (DOCX) [file pone.0227359.s001.docx]

# Data collection tool

# Annex I: Structure questionnaire for patient interview

**Part I. Patients socio-demographic characteristics (Use “X” in the Boxes)**

| Card/code #:_________ | Age:________ | Gender: M F | Pregnancy: Yes No | |
| --- | --- | --- | --- | --- |
| Marital status: | Single | Married | Widowed | Divorced |
| Educational level: | No formal | Primary school | High school | College & above |
| Place of residence | …………………………………………………………………………………. | | | |
| Occupation: | Unemployed | Employed | **d**aily laborer | Merchant |
|  | Student | Farmer | Others---------------------- | |

**Part II Clinical characteristics (supplementary to the information obtained from medical chart)**

1. When did the first seizure episode occur? ____________________________
2. When did you encounter the recent seizure episode? _____________________________
3. How many seizure attacks you encountered in one year period? _______________
4. When did you start your AEDs? _________________
5. How many AEDs you are taking currently? _________________
6. Have you ever encountered an injury due to the seizure? Yes No

If yes specify the type (involved organ) of the injury______________________________

1. Diagnosis other than epilepsy (comorbidity)______________________________________
2. Do you have any medications including non-prescription and traditional medications that you are taking currently? Yes No

If yes please list them _________________________

1. Total number of medications you are taking currently ___________________
2. What is your source of your antiepileptic? medication for free with fee

## Part-III: Assessment of adherence (Morisky Green Levine Test)

| **No** | **Items** | **Yes 1** | **No**  **0** |
| --- | --- | --- | --- |
| **1** | Do you ever forget to take your medicine? | 1 | 0 |
| **2** | Are you careless at times about taking your medicine? | 1 | 0 |
| **3** | When you feel better do you sometimes stop taking your medicine? | 1 | 0 |
| **4** | Sometimes you fell worse, when you take the medicine, do you stop taking it? | 1 | 0 |

**Part IV. Assessment of adverse drug reaction (undesirable effect)**

Have you experienced any undesirable, unusual adverse drug events /allergic reaction to the prescribed medicines? Yes No:

If yes would you describe the manifestation of the events ---------------------------------

| Depression | Head ache | Forgetfulness | Weight gain |
| --- | --- | --- | --- |
| Blurred vision | Gingival hyperplasia | Weakness | Hypersomnia |
| Confusion | Loss of hair | Epigastric pain | Skin rash |
| Irritability | Others please specify----------------------------------------------------- | | |

**Part V. Assessment of Medicine Treatment Satisfaction Questionnaire (SATMED-Q Questionnaire)**

Please write down one of those conditions and answer the questionnaire always referring to the medicine you are using to treat you specific condition and medical condition for which you are taking the medicine. For each question, select the number that best represents your opinion. There are no right or wrong answers. If you are not sure of any of the answers, check the one you consider most appropriate.

|  | Not at all | A little bit | | Some-what | | Quite a bit | Very much | |
| --- | --- | --- | --- | --- | --- | --- | --- | --- |
| **This section is about the side effects of the medicine** |  |  | |  | |  |  | |
| 1. The side effects of the medicine interfere with my physical activities. | 🄋 | ➀ | | ➁ | | ➂ | ➃ | |
| 1. The side effects of the medicine interfere with my leisure and free time activities. | 🄋 | ➀ | | ➁ | | ➂ | ➃ | |
| 1. The side effects of the medicine interfere with my daily activities. | 🄋 | ➀ | | ➁ | | ➂ | ➃ | |
| **This section is about the medicine effectiveness** |  | | | | | | | |
| 1. The medicine I am taking relieves my symptoms. | 🄋 | ➀ | | | ➁ | ➂ | | ➃ |
| 1. I am satisfied with the time it takes for the medicine to start to work. | 🄋 | ➀ | | | ➁ | ➂ | | ➃ |
| 1. I feel better now than I did before starting the treatment. | 🄋 | ➀ | | | ➁ | ➂ | | ➃ |
| **This section is about the convenience and ease of use of the medicine.** |  | | | | | | | |
| 1. I find that taking my medicine is practical for me. | 🄋 | | ➀ | | ➁ | ➂ | | ➃ |
| 1. I find it easy to use/take the medicine in its present form (taste, size, etc.). | 🄋 | | ➀ | | ➁ | ➂ | | ➃ |
| 1. The timetable for taking the medicine suits me. | 🄋 | | ➀ | | ➁ | ➂ | | ➃ |
| **This section is about the impact of the medicine on your everyday life.** |  | | | | | | | |
| 1. Thanks to the medicine I am taking, it is easier for me to do my leisure and free time activities. | 🄋 | | ➀ | | ➁ | ➂ | | ➃ |
| 1. Thanks to my medicine, it is easier for me to take care of my personal hygiene. | 🄋 | | ➀ | | ➁ | ➂ | | ➃ |
| 1. Thanks to my medicine, it is easier for me to perform my daily activities. | 🄋 | | ➀ | | ➁ | ➂ | | ➃ |
| **This section is about the medical follow-up of your condition.** |  | | | | | | | |
| 1. My doctor has informed me in detail about my medical condition. | 🄋 | | ➀ | | ➁ | ➂ | | ➃ |
| 1. My doctor has informed me about the right way to treat my medical condition. | 🄋 | | ➀ | | ➁ | ➂ | | ➃ |
| **general opinion on the medicine and your health** |  | | | | | | | |
| 1. I intend to continue using this treatment. | 🄋 | ➀ | | ➁ | | ➂ | ➃ | |
| 1. I feel comfortable with my treatment. | 🄋 | ➀ | | ➁ | | ➂ | ➃ | |
| 1. In general, I feel satisfied with the treatment. | 🄋 | ➀ | | ➁ | | ➂ | ➃ | |
| **Total** |  | | | | | | | |

# Annex II: Data abstraction format from patient medical records

1. Provisional diagnosis of seizure type _____________________________
2. Diagnosis other than epilepsy (comorbidity)______________________________________
3. Past medical conditions and medications

| Medical condition/ Indication | Drug product (Generic Name) | Dosage regimen (dose, route, frequency, duration) | Date (dd/mm/yy) | | Response Effectiveness/ safety profile |
| --- | --- | --- | --- | --- | --- |
|  |  |  | Started | Stopped |  |
|  |  |  |  |  |  |
|  |  |  |  |  |  |
|  |  |  |  |  |  |
|  |  |  |  |  |  |

1. Physical Examination(PE)/**vital** signs: Consecutive record of visits

| **Parameters** | **Date(dd/mm/yy** |  |  |  |  |  |  |  |  |  |  |
| --- | --- | --- | --- | --- | --- | --- | --- | --- | --- | --- | --- |
| BP |  |  | | |  | | |  |  |  |  |
| PR |  |  | | |  | | |  |  |  |  |
| RR |  |  | | |  | | |  |  |  |  |
| T^0^ |  |  | | |  | | |  |  |  |  |
| Others: |  |  | | |  | | |  |  |  |  |

1. Relevant **laboratory** series results (Findings, at least for three consecutive results).

| **Parameters** | | Date(dd/mm/yy | |  |  |  |  |  |  |  |  |  |  |
| --- | --- | --- | --- | --- | --- | --- | --- | --- | --- | --- | --- | --- | --- |
| Lipid profiles | | Total chol. | |  | | |  | | |  | | |  |
|  |  | LDL: mg/dl | |  | | |  | | |  | | |  |
|  |  | TG: mg/dl | |  | | |  | | |  | | |  |
|  |  | HDL: mg/dl | |  | | |  | | |  | | |  |
|  | | | |  | | |  | | |  | | |  |
| Liver function test | | AST | |  | | |  | | |  | | |  |
|  |  | ALT | |  | | |  | | |  | | |  |
|  |  | ALP | |  | | |  | | |  | | |  |
|  |  | PTT | |  | | |  | | |  | | |  |
|  |  | PT | |  | | |  | | |  | | |  |
|  |  | INR | |  | | |  | | |  | | |  |
|  | | | |  | | |  | | |  | | |  |
| Rena function test | | BUN | |  | | |  | | |  | | |  |
|  |  | Sr. Cr | |  | | |  | | |  | | |  |
|  |  | Cr. Clearance | |  | | |  | | |  | | |  |
|  | | | |  | | |  | | |  | | |  |
| CBC | | WBC | |  | | |  | | |  | | |  |
|  |  | RBC | |  | | |  | | |  | | |  |
|  |  | PLT | |  | | |  | | |  | | |  |
|  |  | Hgb | |  | | |  | | |  | | |  |
|  |  | HCT | |  | | |  | | |  | | |  |
|  |  | MCV | |  | | |  | | |  | | |  |
|  |  | MCHV | |  | | |  | | |  | | |  |
|  |  | Neu. | |  | | |  | | |  | | |  |
|  |  | Lymph. | |  | | |  | | |  | | |  |
|  | | | |  | | |  | | |  | | |  |
| Serum  Electrolyte | | K | |  | | |  | | |  | | |  |
|  |  | Na | |  | | |  | | |  | | |  |
|  |  | Ca | |  | | |  | | |  | | |  |
|  |  | Cl | |  | | |  | | |  | | |  |
| Blood glucose (RBS) or FBS | | | |  | | |  | | |  | | |  |
| Therapeutic drug monitoring if any | | | |  | | |  | | |  | | |  |
| Summary of any other investigations/Diagnostic imaging results | **EEG** | |  | | | | | | | | | | |
|  | **MRI** | |  | | | | | | | | | | |
|  | **CT** | |  | | | | | | | | | | |
|  | **Others** | |  | | | | | | | | | | |

1. Current medical conditions and medications

|  | For the current medical diagnosis (including Comorbid and complications) | | | | |
| --- | --- | --- | --- | --- | --- |
| Medical condition/ Indication | Product name (Generic Name) | Dosage regimen (dose, route, frequency, duration) | Date(dd/mm/yy) | | Response Effectiveness/ safety profile |
|  |  |  | Started | Stopped |  |
|  |  |  |  |  |  |
|  |  |  |  |  |  |
|  |  |  |  |  |  |
|  |  |  |  |  |  |
|  |  |  |  |  |  |

1. Is there any drug interaction ? yes No

If drug interaction is there, specify it ________________________________________________________________________

________________________________________________________________________

1. Was there any experienced adverse effect of the drugs? Yes No

If yes would you describe the manifestation of the events ---------------------------------

| Depression | Head ache | Forgetfulness | Weight gain |
| --- | --- | --- | --- |
| Blurred vision | Gingival hyper plasia | Weakness | Hypersomnia |
| Confusion | Loss of hair | Epigastric pain | Skin rash |
| Irritability | Others please specify----------------------------------------------------- | | |

# Annex III: Modified DTPs Registration Format

| **DTPs Categories** | **Common Cause(s) of Drug therapy problem** |
| --- | --- |
| 1. **Unnecessary drug therapy** | - There is no valid medical indication for the drug therapy at this time Duplicate therapy - Multiple drug products are being used for a condition that requires single drug therapy - The medical condition is more appropriately treated with nondrug therapy - Drug therapy is being taken to treat an avoidable adverse reaction associated with another medication - Drug abuse, alcohol use, or smoking is causing the problem |
| 1. **Needs additional drug therapy** | - Untreated medical condition ( a medical condition requires the initiation of drug therapy) - Preventive/ prophylactic(preventive drug therapy is required to reduce the risk of new condition) - Synergistic/ potentiating (a medical condition requires additional pharmcotherapy to attain synergistic or additive effect) _____________________ |
| 1. **Ineffective drug product** | - The drug product is not the most effective for the indication being treated (In appropriate drug selection) - Condition refractory to drug - Dosage form inappropriate |
| 1. **Adverse drug reaction** | - The drug product causes undesired effect - The drug product is unsafe drug for patient - The drug product causes allergic reactions - The drug product is contraindications due to safety issues - Others, specify_____________________________ |
| 1. **In appropriate dose** | - Dose too high - Dose too low - The duration of drug therapy is too short to produce the desired response |
| 1. **Drug interaction** | - Presence of major drug interaction among the medications |

**ቅጽ 1: የጥናቱ መረጃ ቅጽ (Amharic version(local language) only for the interview part)**

**ክፍል 1. ስለ ታካሚው ማህበራዊ ሁኔታዎች**

| የመለያ ቁጥር#:_________ | ዕድሜ:________ | ፆታ፡  ወ ሴ | እርጉዝ ኖት? አዎ አይደለሁም | | |
| --- | --- | --- | --- | --- | --- |
| የጋብቻ ሁኔታ፡ | ያላገባ/ያላገባች | ያገባ/ያገባች | በሞት የተለየ | | የፈታ/ች |
| የትምህርት ደረጃ: | ያልተማረ/ች | አንደኛ ደረጃ | ሁለተኛ ደረጃ | | ዲፕሎማና ከዛ በላይ |
| መኖሪያ ቦታ፡ | …………………………………………………………………………………. | | | | |
| የስራ ሁኔታ፡ | ስራ አጥ | ተቀጣሪ | የቀን ሰራተኛ | ግልስራ/ነጋዴ | |
|  | ተማሪ | ግብርና | ሌላ/ሌሎች (ይገለፅ) ---------------------- | | |

**ክፍል 2። ከ ህክምናዎ የተያያዘ መረጃ**

1. ይህን ህመምዎት ተመርምረው ካወቁ ምን ያህል ጊዜ ሆኖታል ?-----------------------------

2. ይህን ህመም ለመጨረሻ ጊዜ ካጋጠምዎት ምን ያህል ጊዜ ነው? ----------------------------

3. በዚህ አመት ስንት ጊዜ ህመም አጋጠምዎት? (ይብራራ)---------------------------------------

4. ለዚህ ህመምዎ መድኃኒት መውሰድ ከጀመሩ ምን ያህል ጊዜ ነው; --------------------------

5. ለዚህ ህመምዎ የሚወስዱት የመድሃኒት ብዛት ስንት ነው? (ይዘርዝሩት)------------------------------

6. በዚህ ህመመም ምክንያት ያጋጠምዎት የአካል ጉዳት አለ? አዎ የለብኝም

ካለ እባክዎን ይግልጹት------------------------------------------------------------------------------------- 7. ተጨማሪ ወይም ሌላ ተያያዥ ህመም አለብዎት? አዎ የለብኝም

ካለ እባክዎን ይግልጹት------------------------------------------------

8. ለህመምዎ ከሚወስዱት ሌላ ተጨማሪ ቋሚ መድኃኒቶች ወይም (የባህል ወይም ያለሓኪም ትእዛዝ የሚወሰዱት መድኃኒቶችን) አሉ? አዎ የለም

ካለ እባክዎን ይግልጹት------------------------------------------

9. በአሁኑ ሰአት የሚውሰዱት ጠቅላላ የመድኃኒት አይነት (ብዛት) ስንት ነው?-------------

10. መዴሃኒት የሚያገኙት በምን መሌኩ ነወ? በግዢ በነጻ

**ክፍል 3: ሞሪስኪ” መድኃኒትን በታዘዘው መሰረት በአግባቡ ስለመውሰድ”**

|  | **ጥያቄዎች** | አዎ  1 | አይደለም 0 |
| --- | --- | --- | --- |
| 1 | መድኃኒትዎን ረስተው ሳይወሰዱ ቀርተው ያውቃሉ? | 1 | 0 |
| 2 | ትክክለኛ የመድሃኒት መውሰጃ ሰኣትዎ ላይ ግድ የልሽ ኖት? | 1 | 0 |
| 3 | ህመምዎ ጋብ ሲልሎት (የህመምዎ ስሜቶች ሲጠፉ) አንዳንድ ጊዜ መድኃኒትዎን አቋርጠው | 1 | 0 |
| 4 | ያውቃሉ?መድኃኒትዎን እየወሰዱ ህመምዎ ስላለተሻሎት አንዳንድ ጊዜ መድኃኒትዎን አቋርጠው ያውቃሉ? | 1 | 0 |

**ክፍል 4. ከመድኃኒትዎ የጎኒዮሽ ጉዳት ወይም (ሳይድ ኢፌክት)** **ግምገማን በተመለከተ**

3.1. በአሁን ሰዓት ወይም መድሃኒትዎን መውሰድ ሲጀምሩ ከመድሃኒቱ ጋር የተያያዙ ያልተለመዱ ሁኔታዎች/ የጎኒዮሽ ጉዳት አጋጥሞዎት ያውቃል?

አዎ አላጋጠመኝም

3.2 መልስዎ አዎን ከሆነ የመድሃኒቱን ስሙና የነበረው ሁኔታ ይግለጹ (ከ አንድ በላይ መልስ መምረጥ ይቻላል)

| ድብርት | የራስ ምታት | መርሳት | የክብደት መጨመር |
| --- | --- | --- | --- |
| የዐይን ብዥታ | የድድ ማበጥ | የድካም ስሜት | ከመጠን ያለፈ እንቅልፍ |
| ግራመጋባት | የፀጉር መሳሳት | የጨጓ ራህመም | የቆዳ ላይ ሽፍታና ማሳከክ |
| ራስን ማዞር (ብዥታ) | | ሌላም ካለ ይግፁት------------------- | |

**SATMED-Q ታካሚዎች ስለ ሚወስዱት መድሐኒት/ህክምና ያላቸውን እርካታ መመዘኛ መጠይቅ**

**መመሪያ፡** ለእያንዳንዳቸው ጥያቄ የእርስዎን ስሜት ይበልጥ ይገልጥልኛል ያሉትን ከተሰጡት የእረካታ መመዘኛ አማራጮች መካከል አንዱን ይምረጡ፡፡ ለጥያቄወቹ ትክክለኛ ወይም የተሳሳተ ምላሽ የላቸዉም፡፡ ለሚሰጡት ምላሽ እርግጠኛ ካልሆኑ ይበልጥ ይስማማኛል ብለዉ ያሰቡትን ሀሳብ የያዘልወትነ ምርጫ ይምረጡ፡፡

**በጭራሽ = 0, በትንሹ = 1, በመጠኑ = 2, በጣም = 3 , እጅግ በጣም = 4**

|  |  | በጭራሽ | በትንሹ | በመጠኑ | በጣም | እ. በጣም |
| --- | --- | --- | --- | --- | --- | --- |
| ይህ ክፍል ስለመድሃኒቱ የጎዮሸ ጉዳት ይመለከታል፡፡ | | | | | | |
| 1 | የመድሃኒቱ የጎንዮሽ ጉዳት በአካላዊ እንቅስቃሴ ላይ ተጽእኖ አሳድሯል | 🄋 | ➀ | ➁ | ➂ | ➃ |
| 2 | የመድሃኒቱ የጎንዮሽ ጉዳት በ እረፍት እና በትርፍ ጊዜዬ ላይ ተጽእኖ አሳድሯል | 🄋 | ➀ | ➁ | ➂ | ➃ |
| 3 | የመድሃኒቱ የጎንዮሽ ጉዳት በጠቅለላው የእለት ተእለት እንቅስቃሴዬ ላይ ተጽእኖ አሳድሯል | 🄋 | ➀ | ➁ | ➂ | ➃ |
| ይህ ክፍል ስለ መድሐኒቱ ዉጤታማነት ማለትም በሽታዉን ወይም የበሽታዉን ምልክት ስለማከሙ ይገልፃል፡፡ | | | | | | |
| 4 | የምወስደዉ መድሐኒት የበሽታዬን ምልክቶች አጥፍቶአቸዋል፡፡ | 🄋 | ➀ | ➁ | ➂ | ➃ |
| 5 | መድሐኒቱ ከዋጥኩ በኋላ ቶሎ ለውጥ ስለማይበት ረክቻለሁ፡፡ | 🄋 | ➀ | ➁ | ➂ | ➃ |
| 6 | በፊት ከነበረኝ የጤናሁኔታ ይልቅ ህክምና ከጀመረኩ በኀላ ጥሩ ስሜት እየተሰማኝ ነዉ፡፡ | 🄋 | ➀ | ➁ | ➂ | ➃ |
| ይህ ክፍል ስለመድሐኒቱ ምቹነት ወይም ለአወሳሰድ ቀላል ስለመሆን አለመሆኑ ይመለከታል፡፡ | | | | | | |
| 7 | መድሐኒቶቼ በቀላሉ መወሰድ የሚችሉ እንደሆኑ አዉቂያለሁ፡፡ | 🄋 | ➀ | ➁ | ➂ | ➃ |
| 8 | መድሐኒቶቼ ባሉበት ሁኔታ (በጣዕማቸዉ፣በመጠናቸዉና በመሳሰሉት) በቀላሉ መዉሰድ እንደምችል አዉቂያለሁ፡፡ | 🄋 | ➀ | ➁ | ➂ | ➃ |
| 9 | መድሐኒቶቼን የምወስድበት የጊዜ ሰሌዳ ተመችቶኛል፡፡ | 🄋 | ➀ | ➁ | ➂ | ➃ |
| ይህ ክፍል መድሐኒቱ በዕለትከዕለት ኑሮዎት ላይ ስለሚኖረዉ ሚና ይመለከታል፡፡ | | | | | | |
| 10 | ዕድሜ ለምወስደዉ መድሐኒት በትርፍ ጊዚዬ የምሰራቸዉን ስራዎች ማከናዎን ቀላል ሆኖልኛል ነዉ፡፡ | 🄋 | ➀ | ➁ | ➂ | ➃ |
| 11 | ዕድሜ ለመድሐኒቴ የግል ንፅህናዬን ለመጠበቅ ቀላል ሆኖልኛል ነዉ፡፡ | 🄋 | ➀ | ➁ | ➂ | ➃ |
| 12 | ዕድሜ ለመድሐኒቴ የእለትከእለት እንቅስቃሴዬን ለማከናወን ቀላል ሆኖልኛል ነዉ፡፡ | 🄋 | ➀ | ➁ | ➂ | ➃ |
| ይህ ክፍል ስለህክምናዉ ክትትል ይመለከታል፡፡ | | | | | | |
| 13 | ሐኪሜ ስለጤናዬ ሁኔታ በጥልቀት አሳዉቆኛል፡፡ | 🄋 | ➀ | ➁ | ➂ | ➃ |
| 14 | ሐኪሜ ያጋጠመኝ የጤናችግር በተገቢዉ ሁኔታ እንዴት መታከም እንዳለበት አሳዉቆኛል፡፡ | 🄋 | ➀ | ➁ | ➂ | ➃ |
| በመጨረሻም መጠይቁ ስለመድሐኒቱና ስለታካሚዉ ጤንነት አጠቃላይ ያለዉን ሃሳብ ምን እንደ ሚመስል ይመለከታል፡፡ | | | | | | |
| 15 | መድሐኒቱን በቀጣይነት ለመዉሰድ አቅጃለሁ | 🄋 | ➀ | ➁ | ➂ | ➃ |
| 16 | በሚደረግልኝ ህክምና ምቾት ተሰምቶኛል፡፡ | 🄋 | ➀ | ➁ | ➂ | ➃ |
| 17 | በአጠቃላይ በሚደረግልኝ ህክምና ረክቻለሁ፡፡ | 🄋 | ➀ | ➁ | ➂ | ➃ |
|  | ጠቅላላ ውጤት |  | | |  |  |
